# Identification of Nitrogen Starvation-Responsive miRNAs to Reveal the miRNA-Mediated Regulatory Network in *Betula luminifera*

**DOI:** 10.3389/fgene.2022.957505

**Published:** 2022-08-17

**Authors:** Yan Lin, Sasa Chu, Xiaoshan Xu, Xiao Han, Huahong Huang, Zaikang Tong, Junhong Zhang

**Affiliations:** State Key Laboratory of Subtropical Silviculture, School of Forestry & Bio-technology, Zhejiang A&F University, Hangzhou, China

**Keywords:** miRNA, nitrogen starvation, target genes, Betula luminifera, sRNA sequencing

## Abstract

Because of the immobility, plants encounter a series of stresses, such as varied nutrient concentrations in soil, which regulate plant growth, development, and phase transitions. Nitrogen (N) is one of the most limiting factors for plants, which was exemplified by the fact that low nitrogen (LN) has a great adverse effect on plant growth and development. In the present study, we explored the potential role of microRNAs (miRNAs) in response to LN stress in *Betula luminifera*. We identified 198 miRNAs using sRNA sequencing, including 155 known and 43 novel miRNAs. Among them, 98 known miRNAs and 31 novel miRNAs were differentially expressed after 0.5 h or 24 h of LN stress. Based on degradome data, 122 differential expressed miRNAs (DEmiRNAs) including 102 known miRNAs and 20 novel miRNAs targeted 203 genes, comprising 321 miRNA–target pairs. A big proportion of target genes were transcription factors and functional proteins, and most of the Gene Ontology terms were enriched in biological processes; moreover, one Kyoto Encyclopedia of Genes and Genomes term “ascorbate and aldarate metabolism” was significantly enriched. The expression patterns of six miRNAs and their corresponding target genes under LN stress were monitored. According to the potential function for targets of DEmiRNAs, a proposed regulatory network mediated by miRNA–target pairs under LN stress in *B. luminifera* was constructed. Taken together, these findings provide useful information to elucidate miRNA functions and establish a framework for exploring N signaling networks mediated by miRNAs in *B. luminifera*. It may provide new insights into the genetic engineering of the high use efficiency of N in forestry trees.

## Introduction

Nitrogen (N) is one of the most indispensable elements for plant growth and development, which is considered the main limiting macronutrient (Perez-Tienda et al., 2014). N participates in a variety of biological processes, such as protein synthesis, nucleic acid synthesis, chlorophyll synthesis, ATP synthesis, and phytohormone synthesis, and acts as a signal (Zhao et al., 2015). Nevertheless, the majority of soil does not satisfy the plant’s needs (Perez-Tienda et al., 2014). Although a large amount of fertilizers are applied, relatively low efficiency for fertilizer is commonly observed, leading to environmental pollution and even harm to human health (Ahmed et al., 2017). Therefore, when a plant encounters N starvation, it is urgent to understand the molecular regulatory mechanism of adaption. Plants often grow in barren soil with limited N, especially forestry trees that are usually planted in infertile soil. *Betula luminifera* with rapid growth and excellent wood properties, as a pioneer tree, often grows in barren soil with limited N source, which restricts significantly the growth and productivity. It is urgent to elucidate the adaption mechanism of N starvation in this forestry tree species, which might help to make breeding strategies improve tolerance and adaptation to N starvation in *B. luminifera* and other forestry trees.

Amounting evidence showed that plants have evolved a complex but systematic adaptive mechanism to adapt the N starvation, such as gene transcription and regulation (Liu et al., 2017; Ye et al., 2019), morphological changes (Wang et al., 2019), and photosynthetic characteristics and metabolic pathways (Liu et al., 2019; Zhao et al., 2020). Among them, increasing studies suggest that miRNAs play an important role in response to N starvation, by regulating the expression of target genes, triggering the change of physiological and biochemistry characteristics in many species (Zuluaga and Sonnante, 2019; Li L. J. et al., 2021).

miRNA is a class of 18–24-nt endogenous non-coding RNA, which is processed from the *MIRNA* gene with stem-loop structure by Dicer-like (DCL) (Jones-Rhoades et al., 2006). Plant miRNAs are usually highly complementary with their target genes, triggering the target mRNA cleavage by RNA-induced silencing complex. However, the translational inhibition is occurred in the target genes, when the seed region of miRNAs harbors relatively low complementary with target mRNA (Brodersen et al., 2008). In 2002, miRNA was first found in plants, which was proved to exert various effects on plants (Llave et al., 2002). Previous studies showed that miRNAs are shown to take part in the regulation of growth and development, as well as acclimatization to adversity stress. For instance, miR396e and miR396f are two important regulators of grain size and plant architecture in rice (Miao et al., 2020). The upregulated miR160, miR167, and miR393 might be participated in the regulation of primary root length and lateral root number under N deficiency (Li L. J. et al., 2021). Cui et al. (2020) showed that miR1885 dynamically regulates both innate immunity and plant growth and responds to viral infection, by targeting both TIR-NBS-LRR and a photosynthesis-related gene for negative regulation through distinct modes of action (Cui et al., 2020). Amounting studies have demonstrated that miRNAs help plants to adapt to drought stress (Bhat et al., 2020), cold stress (Yang et al., 2017), heat stress (Pan et al., 2017), and heavy metal toxicity (Celik and Akdas, 2019). In recent years, several studies showed that miRNA–target modules played important roles in response to nutritive stress. For instance, the miR396e/f-GRF4/6/8 module takes part in the regulation of yield, N assimilation, and utilization in rice, and mir396e/f mutants have higher N content and yield in N-deficient conditions (Zhang et al., 2020). TamiR2275 was induced by N starvation stress, which regulated N acquisition and photosynthetic function when plants are challenged by N deprivation (Qiao et al., 2018)*.* Our previous study demonstrated that a potential regulatory mechanism of the miR169c-*NFYA10* module is involved in the low-nitrogen (LN) stress response of *B. luminifera* (Cheng et al., 2021). However, the expression profiles of miRNAs in response to LN stress are waiting to be elucidated in *B. luminifera*.

In recent years, the widespread high-throughput sequencing strategy was used for sRNAome (Wen et al., 2022). The ability to detect low-expressed miRNAs contributes to the tremendous progress of miRNAs, especially in non-model plant species (Dos Santos et al., 2019; Li H. et al., 2021). Furthermore, the combination of sRNA sequencing and degradome sequencing promotes the identification of miRNA target genes (Charles Addo-Quaye, 2008). In our previous study, we identified 114 known miRNAs and defined 49 targets for 26 miRNA families in *B. luminifera* (Zhang et al., 2016), and 44 miRNAs and 71 corresponding target genes were identified in response to heat stress (Pan et al., 2017). Therefore, in the present study, we sequenced and analyzed sRNA libraries and roots and shoots under control, 0.5 h N-starved, and 24 h N-starved treatments, respectively. We tested two hypotheses: 1) The abundance of miRNAs is affected by N starvation, and these miRNAs might have regulatory roles in N starvation adaptation in *B. luminifera* by regulating the abundance of their corresponding target genes. 2) The expression patterns of N starvation-responsive miRNAs change with stress duration.

## Materials and Methods

### Plant Cultivation and Low-Nitrogen Treatments

The *B. luminifera* G49^#^ seedlings were propagated *in vitro* in 1/2 MS at the temperature of 25 ± 2°C with a 16-h photoperiod. After 2-month growth, seedlings were transferred to a mixed substance consisting of perlite, peat, and clay loam in proportion to 1:1:2, under 120 μmol m^−2^•s^−1^ light for 2 weeks. Then, 27 uniformly growing individuals were selected and randomly divided into two groups, namely, CK and LN. Seedlings in the CK group were cultivated using half-strength liquid MS media including 15 mm KNO_3_, whereas the seedlings in the LN group were cultivated using half-strength MS media containing 0.03 mm KNO_3_ (the only source of N), as described previously (Cheng et al., 2021). Then, the leaves and roots were sampled individually after 0.5 and 24 h after the CK and LN treatments, at 11:00 a.m., respectively. Three randomly selected seedlings were pooled together as one replicate, with three replicates per group, and samples were flash frozen in liquid N and stored at −80°C until RNA extraction.

### The Expression Patterns of Low-Nitrogen-Responsive Marker Genes

Total RNA was extracted using TRIZOL (Invitrogen, Carlsbad, CA, United States) and was then reverse transcribed to cDNA using PrimeScript RT Reagent Kit (TaKaRa, Dalian, China). To verify the effectiveness of LN stress treatment, two marker genes *NRT2.5* and *NRT2.7* were quantified via qRT-PCR (Supplementary Table S1). The large ribosomal subunit 39 (*RPL39*) was used as a reference gene to normalize the expression levels of marker genes (Cheng et al., 2021).

### sRNA Library Construction, Sequencing, and Bioinformatics

The construction and sequencing of the sRNA library were accomplished by LC Bio (Hangzhou, China) with Illumina Hiseq 2500. After the quality assessment of sequencing data, the retained data were processed using ACGT101-miR (LC Sciences, Houston, Texas, United States), including removing adaptor dimers, junk sequences, and low complexity sequences and filtering out mRNA, Rfam, and Repbase. Then, the remained sequences were blasted against miRBase21.0 and genome, and the hairpin structure of predicted miRNA precursor sequences was validated using RNAfold software (http://rna.tbi.univie.ac. at/cgi-bin/RNAfold. Cgi).

### Analysis of Differentially Expressed miRNAs

The reads of miRNAs from the control, 0.5 h, and 24 h of LN treatment were normalized to tags per million. The combination of log2 ratio and Fisher’s exact test was used to identify the differential expressed miRNAs (DEmiRNAs). Only when the absolute value of log2 ratio ≥ 1 and *p* ≤ 0.05, the miRNA was significantly differentially expressed; however, in other cases, there was no significant difference in expression (Xie et al., 2015).

### Target Prediction of DEmiRNAs and Functional Classification

Target prediction of DEmiRNAs both known and novel miRNAs was performed using Targetfinder (http://targetfinder.org/), and the mismatch score was less than four. Based on our published degradome sequencing data of *B. luminifera* (GEO: GSE80074), we used CleaveLand 3.0.1 pipeline to identify and classify the target genes of DEmiRNAs. To further elucidates the potential regulation of target genes, GO (Gene Ontology) analysis was conducted using agriGO2.0 (http://bioinfo.cau.edu.cn/agriGO/). In addition, Kyoto Encyclopedia of Genes and Genomes (KEGG) annotation was conducted using a database (http://www.genome.jp/kegg/kegg1.html).

### The Expression Patterns of miRNAs and Their Target Genes

Total RNA was extracted as described above and was reversely transcribed with Mir-X miRNA First-Strand Synthesis Kit (TaKaRa, Dalian, China). qRT-PCR was conducted using SYBR Premix EX Taq Kit (TaKaRa). Gene *U6* was used as a reference gene to normalize the expression levels of miRNAs. The RNA was reversely transcribed to cDNA using PrimeScript RT Reagent Kit (TaKaRa), which was used to quantify the target genes. The *RPL39* was used as a reference gene, and the relative expression levels of genes were calculated *via* the 2^−ΔΔCt^ method. The qRT-PCR primers are shown in Supplementary Table S1.

## Results

### The Expression Patterns of Low-Nitrogen-Responsive Marker Genes in *B. luminifera*

**FIGURE 1:**
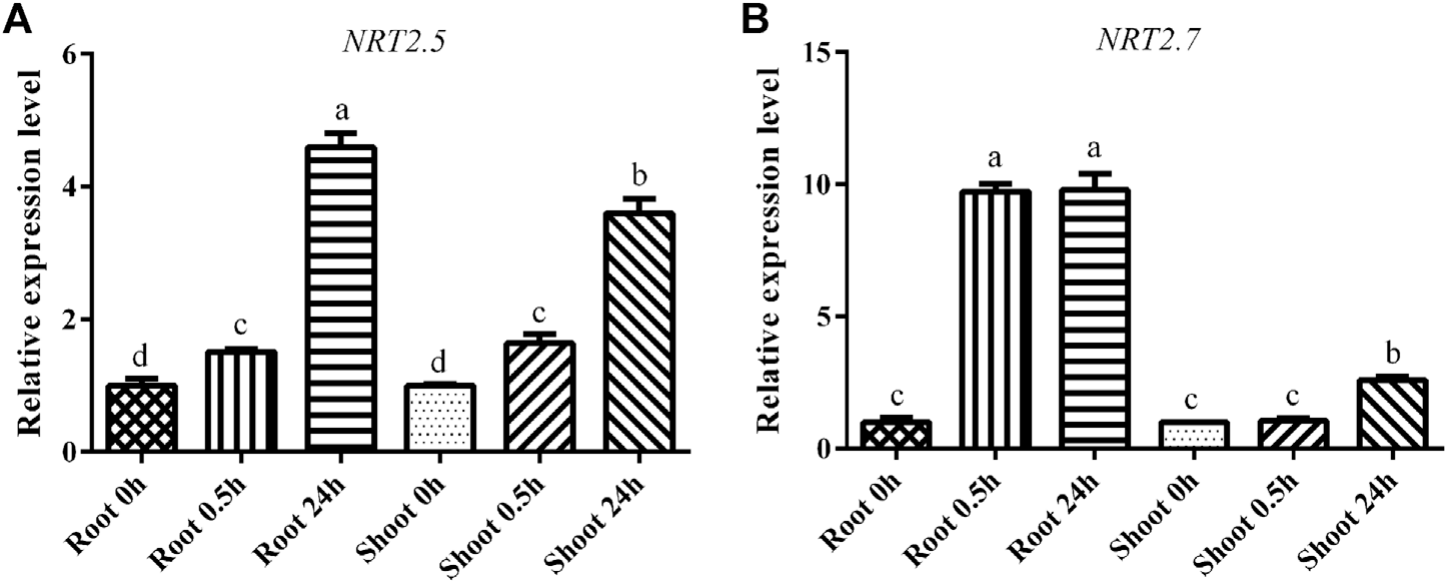
The expression levels of marker genes under low nitrogen (LN) treatment. **(A)** The expression level of *BlNRT2.5*; **(B)** The expression level of *BlNRT2.7* under LN treatment. The reference gene was *BlRPL39*, each sample had three replicates, and error bars were standard deviation.

To confirm the physiological status of the *B. luminifera* seedlings (i.e., to confirm whether the plants were subjected to LN stress), the expression levels of the marker genes *BlNRT2.5* and *BlNRT2.7* were analyzed. *BlNRT2.5* and *BlNRT2.7* as HATS, responsible for nitrate transport and absorption under LN, are indicators of whether the plants significantly underwent LN stress, as reported previously (Wang et al., 2012). In both root and shoot, the expression level of *BlNRT2.5* was increased significantly after 0.5 h of LN treatment and reached the peak value after 24 h of LN treatment (Figure 1A). For the *BlNRT2.7*, the expression level was induced 10-fold after 0.5 h of LN treatment and remained high level after 24 h of N starvation in the root, whereas the expression level was unchanged until 24 h of LN treatment in the shoot (Figure 1B).

### sRNA Profiles in Response to Low-Nitrogen Stress

To investigate the response of miRNAs to N starvation, six mixed sRNA libraries (each library was a pool of RNAs from three biological samples) were generated from roots and shoots under LN stress for 0, 0.5, and 24 h, respectively, and then were sequenced via Solexa high-throughput sequencing technology. After the remove of adaptor and junk sequences, a total of 66,031,208 high-quality reads were obtained and then mapped to known RNAs, resulting in 2,305,703 (461,778), 5,012,446 (1,028,698), 2,663,870 (514,935), 3,475,390 (785,402), 4,953,286 (1,468,323), and 1,322,911 (319.385) redundant (unique) valid reads, which were used to identify miRNAs (Supplementary Table S2).

### Length Distribution of sRNAs Under CK and Low-Nitrogen Conditions

**FIGURE 2:**
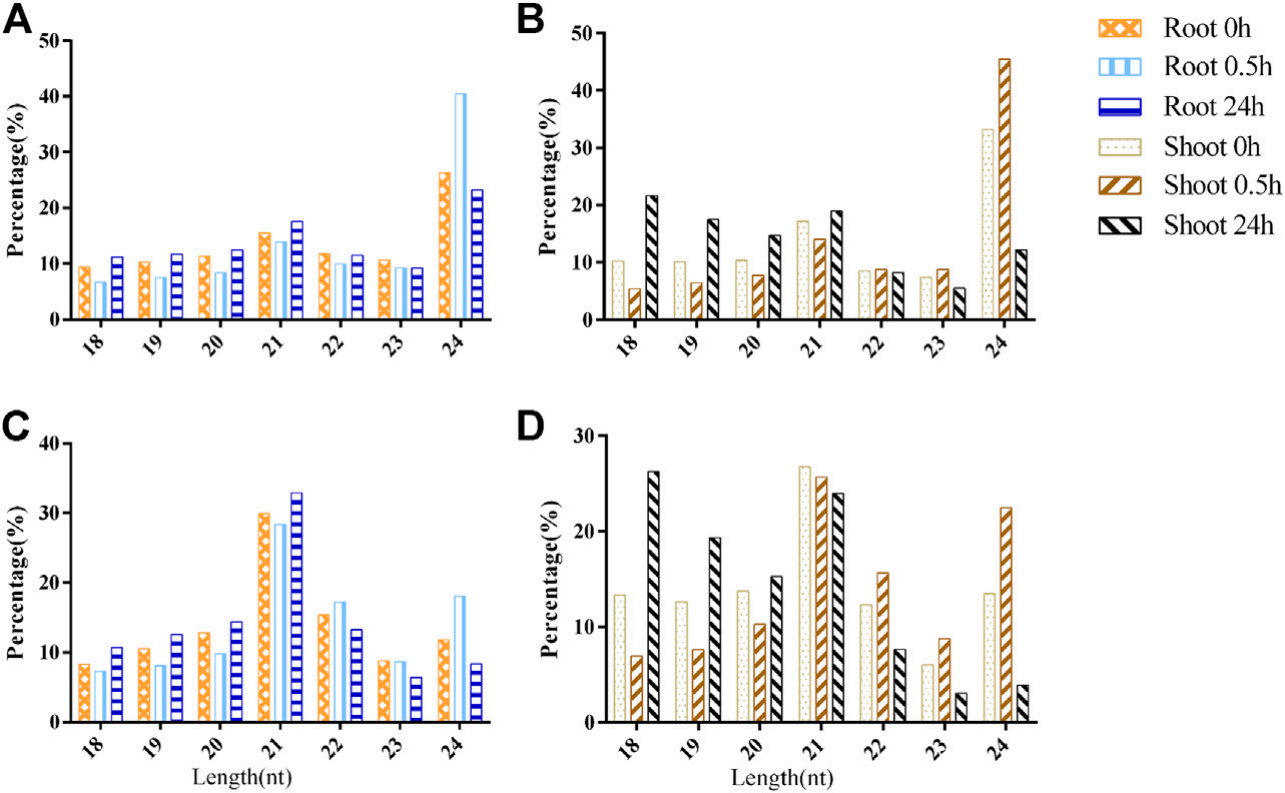
The length distribution of sRNAs under low-nitrogen stress. **(A)**. Unique valid reads in root; **(B)**. Unique valid reads in shoot; **(C)**. Redundant valid reads in root; and **(D)**. Redundant valid reads in shoot.

Most of the sRNA sequences ranged from 18 to 24 nt, representing 97.87% (96.58%) of redundant (unique) valid reads, which was consistent with the characteristic of the DCL1 cleavage product. In the root, 24-nt sRNAs had the highest proportion of sRNAs in the unique valid reads (Figure 2A). Under LN stress, the length distribution patterns of sRNAs in the root were altered, which was exemplified by the fact that the ratio of 24-nt sRNAs increased from 26.31% (0 h) to 40.42% (0.5 h) and then recovered to 23.24% (24 h). A similar phenomenon was observed in the shoot, which was exemplified by the fact that 24-nt sRNAs accounted for 32.26% in 0 h and increased to 45.49% after 0.5 h of LN stress and then reduced to 12.20% (Figure 2B). However, the 18-nt sRNAs had the highest proportion, accounting for 21.66% in the shoot after 24-nt LN stress.

For redundant valid reads, 21-nt sRNAs were the most abundant class of sRNAs in the root, and the ratio of 24-nt sRNAs increased after 0.5 h of LN treatment (Figure 2C). In the shoot, 21-nt sRNAs were also the most abundant class, accounting for 26.76 and 25.69% in the 0 and 0.5 h of LN treatment, respectively. However, the ratio of 18-nt sRNAs reached the peak level after 24 h of LN stress (Figure 2D).

### Identification of Known and Novel miRNAs in *B. luminifera*

A total of 155 known miRNAs from 55 families were identified, and 43 novel miRNAs from 32 families were discovered, resulting in 198 miRNAs from 87 families. Among them, 113 known miRNAs were identified with according precursors, whereas other 42 known miRNAs lacked the corresponding precursors, and miR166 was the most abundant miRNA family (Supplementary Table S3).

### miRNA Expression Response to Low-Nitrogen Stress

**FIGURE 3:**
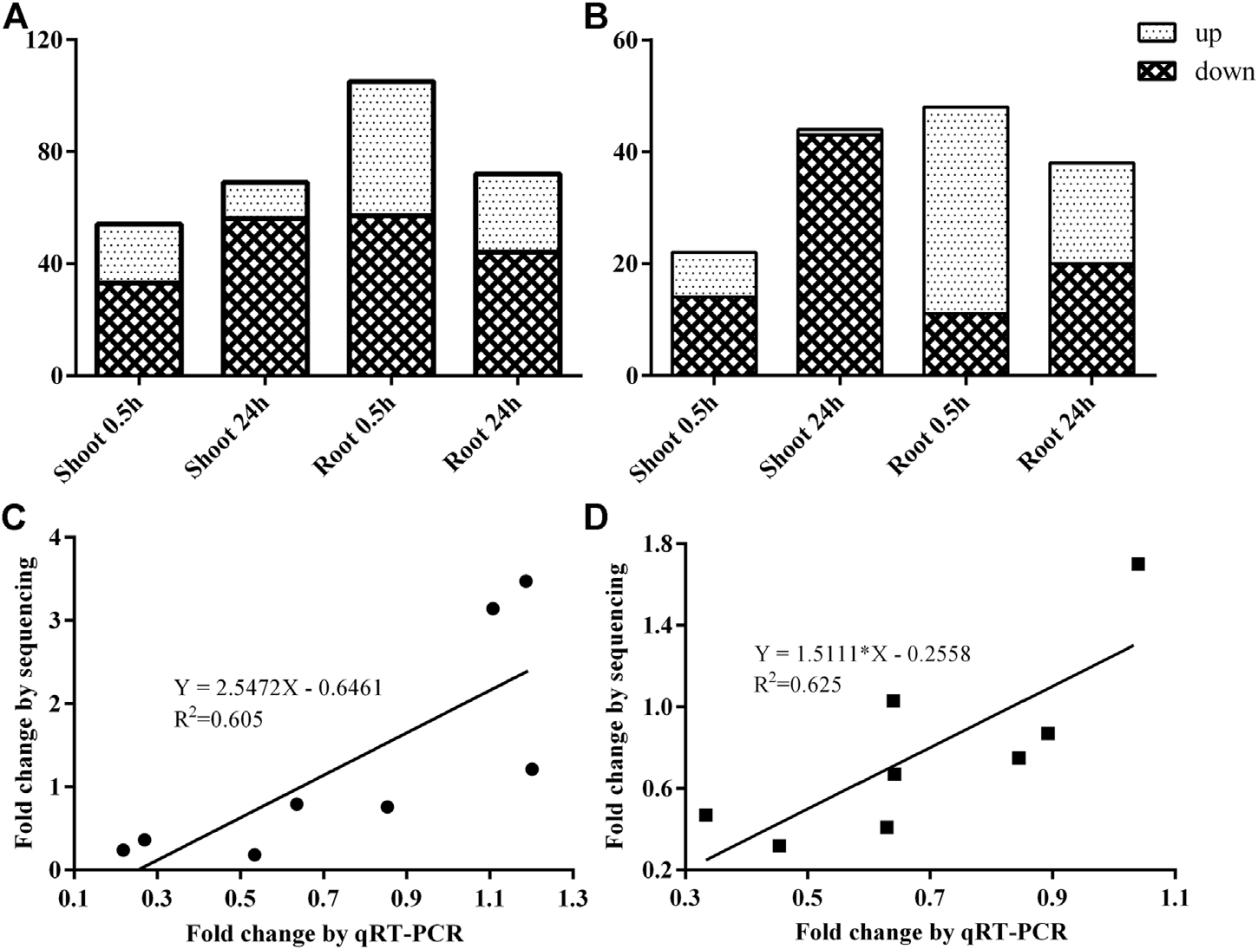
The identification and validation of DEmiRNAs after 0.5 and 24 h N-starved treatment. **(A)** Total number of DEmiRNA in shoot and root; **(B)** The specific DEmiRNA in shoot and root; **(C)** The validation of DEmiRNAs after 0.5 h LN treatment; and **(D)** The validation of DEmiRNAs after 24 h of low nitrogen treatment.

In the shoot, 76 known miRNAs from 42 families and 22 novel miRNAs from 20 families were recognized as DEmiRNAs after 0.5 h and/or 24 h N-starved treatment, whereas 98 known miRNAs from 46 families and 31 novel miRNAs from 24 families were differentially expressed in the root (Supplementary Table S4). To be precise, 54 and 69 DEmiRNAs were identified in the shoot, whereas 105 and 72 DEmiRNAs were detected in the root after 0.5 and 24 h N-starved treatment, respectively (Figure 3A). Among them, 22 and 44 DEmiRNAs were specifically expressed in the shoot after 0.5 and 24 h treatment, respectively, whereas 48 and 38 DEmiRNAs were specifically identified in the root (Figure 3B, Supplementary Table S4).

To validate the results of the sRNA sequencing, eight DEmiRNAs were selected randomly for qRT-PCR analysis. Although the fold-changes of DEmiRNAs between qPCR and sRNA sequencing were different, similar expression tendencies were observed (Figures 3C,D).

### Target Genes Regulated by DEmiRNA

**FIGURE 4:**
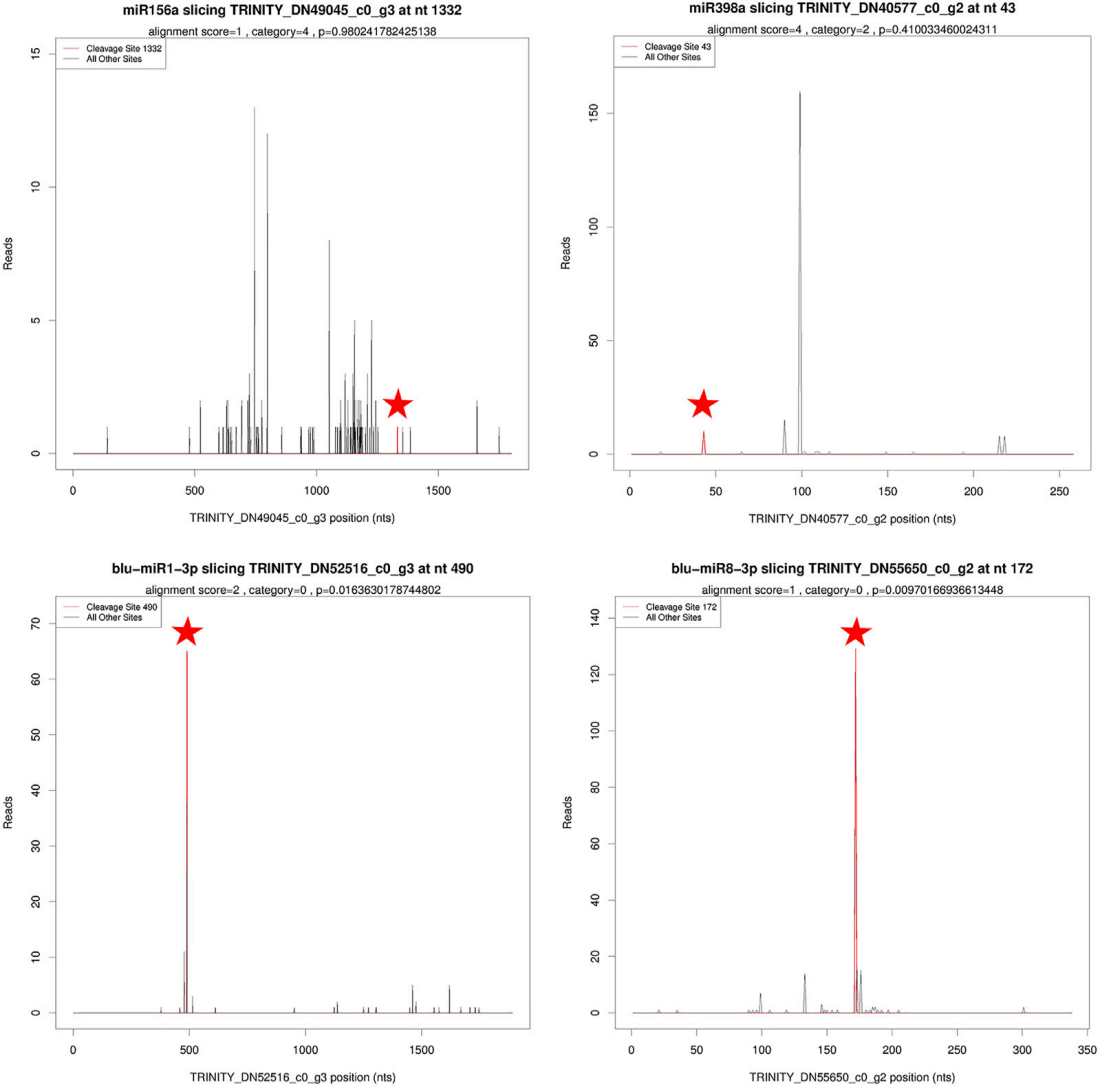
T-plots of miRNA targets based on degradome sequencing. Asterisks were used to mark cleavage sites of target genes.

A total of 167 miRNAs were predicted to target 836 genes, involving 1,094 miRNA–target pairs. Based on degradome data, 122 miRNAs including 102 known miRNAs and 20 novel miRNAs targeted 203 genes, comprising 321 miRNA–target pairs (Supplementary Table S5). These targets were divided into five categories: categories 0–4 (Figure 4). For known miRNAs, most of the target genes were annotated as transcription factors. For instance, *SPL13A* (Squamosa promoter-binding protein-like, *SPL*) was targeted by three miR156, *NAC021* (NAM, ATAF, CUC, *NAC*) by miR164a, *NFYA2/7* (*Nuclear* Transcription Factor Y Subunit A, *NFYA*) by five miR169, *RAP2–7* (Ethylene-responsive transcription factor, *RAP*) by miR172a/b, and *GRF4/6* (Growth-Regulating Factor, *GRF*) by miR396a/b. A big proportion of target genes for novel miRNAs were proteins or enzymes. For example, *NSP2* (Nodulation-Signaling Pathway 2 protein) was targeted by blu-miR1-3p, *CMTA2* (CalModulin-binding Transcription Activator 2-like) by blu-miR5-3p, *OPT3* (OligoPeptide Transporter 3) by blu-miR7-3p, and FBX (F-box/kelch-repeat protein) by blu-miR8-3p.

### Gene Ontology and Kyoto Encyclopedia of Genes and Genomes Pathway Analyses of Target Genes

**FIGURE 5:**
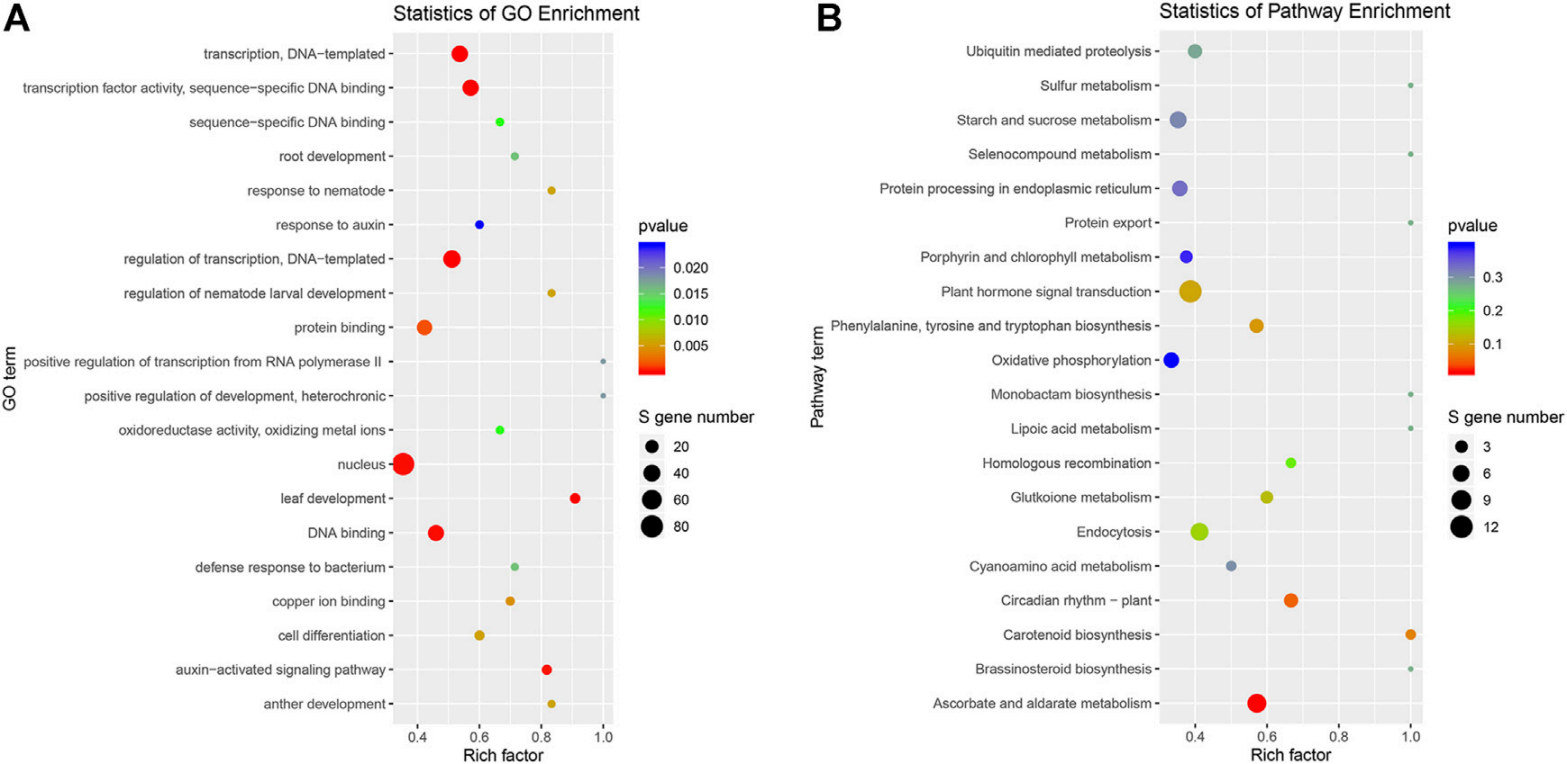
Gene ontology (GO) and Kyoto Encyclopedia of Genes and Genomes (KEGG) pathway enrichment analyses of genes targeted by DEmiRNAs. **(A)** GO enrichment analysis; **(B)** KEGG pathway enrichment analysis of genes targeted by DEmiRNAs.

GO was used to predict the function of target genes. A total of 665 target genes were annotated with 1,115 GO terms (Supplementary Table S6). Among them, a large proportion (54.08%) of target genes were classified into biological processes, one-third of genes for molecular function (327 terms), and 169 items belonging to cellular components. Of these, 13 GO terms were extremely significantly enriched (*p* < 0.01), including leaf development, auxin-activated signaling pathway, cell differentiation, transcription factor activity, DNA binding, and nucleus (Figure 5A). One KEGG term “ascorbate and aldarate metabolism” (ko00053) was significantly enriched (*p* < 0.05) (Figure 5B).

### Expression Patterns of miRNAs and Their Target Genes Under Low-Nitrogen Stress

**FIGURE 6:**
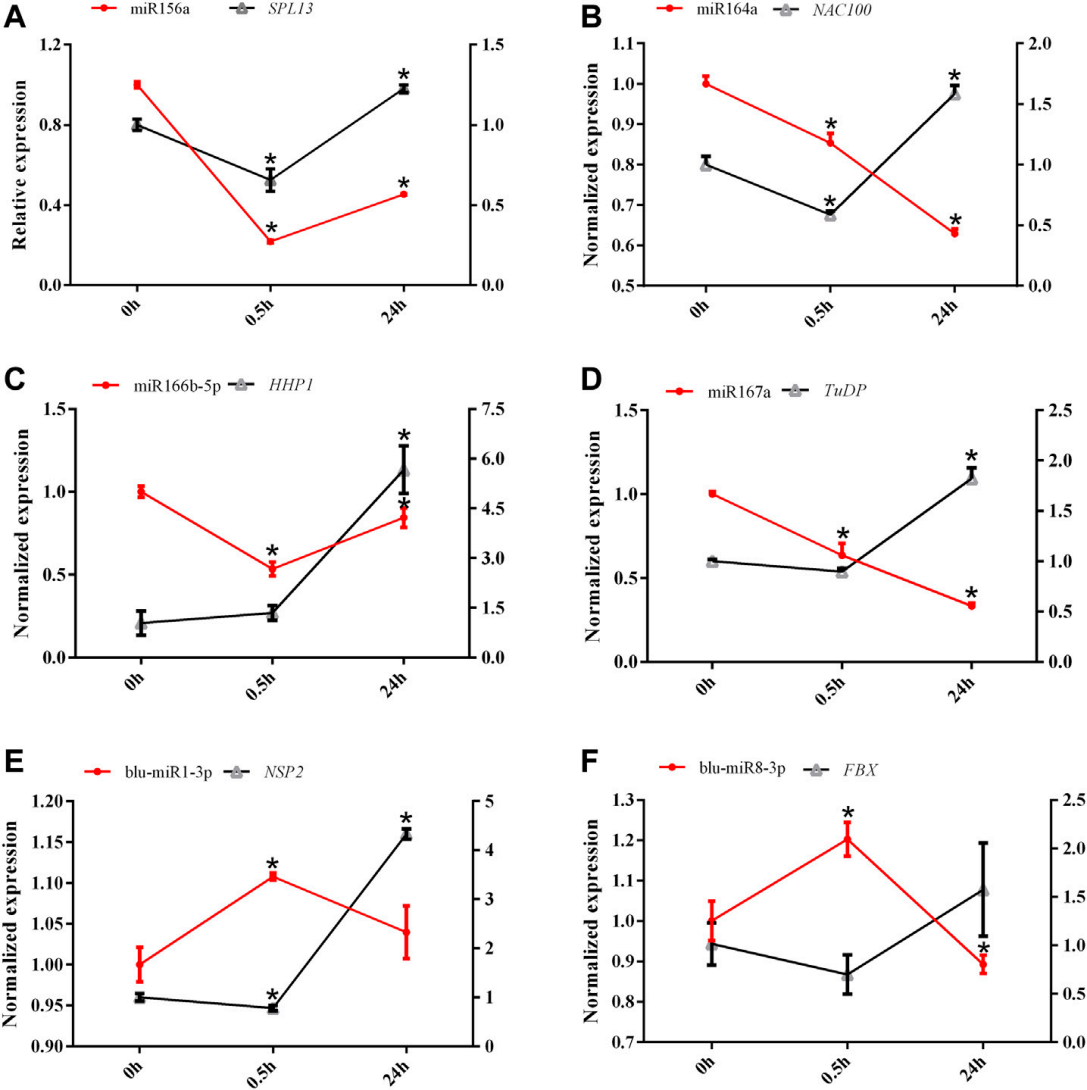
The expression levels of six miRNAs and their predicted target genes under low-nitrogen stress. The left *y* axis represents the expression level of miRNA, and the right *Y* axis represents the expression level of the predicted target gene. All expression was normalized by *BlRPL39*, and three replicates were conducted. **(A)** miR156a-*SPL13*; **(B)** miR164a-*NAC100*; **(C)** miR166b-5p-*HHP1*; **(D)** miR167a-*TuDP*; **(E)** miR1-3p-*NSP2*; **(F)** miR8-3p-*FBX*.

The expression patterns of six miRNAs and their target genes were monitored after 0.5 and 24 h of LN treatment (Figure 6). The expression levels of miR156a and miR166b-5p were initially decreased after 0.5 h of N starvation but increased after 24 h of LN treatment, whereas the expression patterns of blu-miR1-3p and blu-miR8-3p showed an opposite trend. However, miR164a and miR167a exhibited continuous decreasing expression patterns under LN treatment. To determine the relationships between miRNAs and target genes, we examined the expression levels of target genes under LN treatment using qRT-PCR (Figure 6). The overall expression levels of target genes were unchanged or decreased slightly after 0.5 h of LN stress but were induced significantly by 24 h of LN stress.

## Discussion

N is one of the most important restrictive elements in plant growth and development, and nitrate is the major form for most land plants (Glass et al., 2002). The limited nitrate in soil restricts plant growth; thus, it is of great importance to understand how plants respond to nitrate deficiency. Amounting studies showed that miRNAs played important roles in response to N starvation, such as *Arabidopsis* (Zhao et al., 2011), wheat (Liu et al., 2021), potato (Tiwari et al., 2020), and rice (Yu et al., 2018).

### Length Distribution of sRNAs Varied Under Low-Nitrogen Stress

Several plant species, such as *Arabidopsis*, cotton, and Chinese white poplar, the most redundant sRNAs, were 21-nt sized. In the present study, 21-nt sRNAs were the most abundant class of sRNAs in both root and shoot of *B. luminifera* (Figures 2C,D). A similar phenomenon was observed in our previous study (Zhang et al., 2016), whereas the 24-nt class of sRNAs was the most dominant in heat-stressed *B. luminifera* (Pan et al., 2017). It might be attributed to developmental- and/or stress-specific differences. In the *Chrysanthemum nankingense*, 21- and 24-nt sRNAs were the most abundant classes, and the proportion of 24-nt sRNA in the root was higher than that of 21-nt sRNA, but it was the opposite in leaves (Song et al., 2015). In potatoes, 24-nt sRNAs accounted for the largest proportion in all classes, followed by 21-nt sRNAs (Tiwari et al., 2020). In maize, the proportion of 21-nt sRNA was the highest under all treatments of shoots, which was the same in the root under control treatment, but the sRNA of the root was mainly distributed in 24 and 22 nt under LN treatment (Zhao et al., 2012). Moreover, the ratio of 24-nt sRNAs increased in the root after 0.5 h of LN treatment (Figure 2C), and the ratio of 18-nt sRNAs reached the peak level after 24 h of LN stress in the shoot (Figure 2D). It was consistent with the major peak at 18 nt in *B. luminifera* roots under LP stress (Zhang et al., 2021). A previous study showed that the components of sRNAs with the size of 18–19 nt originated from tRNAs, and the accumulation of tRNA fragments in Pi-starved (Hsieh et al., 2009) and nitrate-starved *Arabidopsis* (Liang et al., 2012). Moreover, tRNA halves were detected in the phloem sap of *Cucurbita maxima*, which inhibited translational activity *in vitro*, indicating that these tRNA halves coordinate the metabolic status between source and sink tissues, acting as long-distance signals (Zhang et al., 2009). Loss-Morais et al. (2013) showed that tRNA fragments can be incorporated into argonaute (AGO) complexes, thus regulating gene expression post-transcriptionally, acting as miRNAs (Loss-Morais et al., 2013). Therefore, these 18-nt sRNAs induced by LN stress might play important regulatory roles in response to N starvation in *B. luminifera*, which need to be further studied.

### miRNA Expression Response to Nitrogen Starvation

Previous studies have identified a series of LN-responsive miRNAs. For instance, 102 miRNAs from 42 families in maize (Zhao et al., 2012) and 404 and 628 putative miRNAs were identified in potato roots and shoots, respectively (Tiwari et al., 2020). Although three studies on miRNA of *B. luminifera* have been conducted (Zhang et al., 2016; Pan et al., 2017; Zhang et al., 2021), the LN-responsive miRNAs were waiting to be identified. In the present study, a total of 198 miRNAs from 87 families were identified, of which 113 known miRNAs were identified with precursors. Thus, it enriches the miRNA information of *B. luminifera*.

miR166 was the most abundant miRNA family, and miR166a accounted for the largest proportion, which was consistent with the result of Chinese cabbage (Ahmed et al., 2019), whereas miR166a-3p was the most abundant miRNA in our previous study (Pan et al., 2017). The inconsistency might relate to the alternative cleavage mechanism depending on the plant species, growth stage, and/or stress. For example, the miR535 family had the highest abundance, and miR156, miR166, and miR168 families also had high expression levels in bananas (Zhu et al., 2019). Several miRNAs were specifically expressed in some samples (Supplementary Table S4), such as 156 d-3p in a 0.5 h LN-treated shoot. A similar phenomenon was observed in potatoes, where five conserved miRNAs were specifically expressed in roots and nine in shoots. It indicated that the accumulation of some miRNAs is dependent on plant species, tissues, and/or stresses. In the present study, more downregulated DEmiRNAs were observed under LN stress (Figure 3). A similar phenomenon was observed in sorghum and durum wheat, which was exemplified by the fact that the number of downregulated DEmiRNAs was more than that of upregulated DEmiRNAs under LN stress (Liu et al., 2021; Zhu et al., 2021). The GO analysis of DEmiRNA targets showed that leaf development, auxin-activated signaling pathway, cell differentiation, and transcription factor activity were enriched (Figure 5A), and the KEGG term “ascorbate and aldarate metabolism” (ko00053) was significantly enriched (Figure 5B). It suggested that miRNAs play important roles in leaf development, hormone signaling pathway, cell differentiation, ascorbate and aldarate metabolism, and transcriptional regulations in response to LN stress in *B. luminifera*.

### Expression Patterns of miRNAs and Targets in *B. luminifera* Under Low-Nitrogen Stress

**FIGURE 7:**
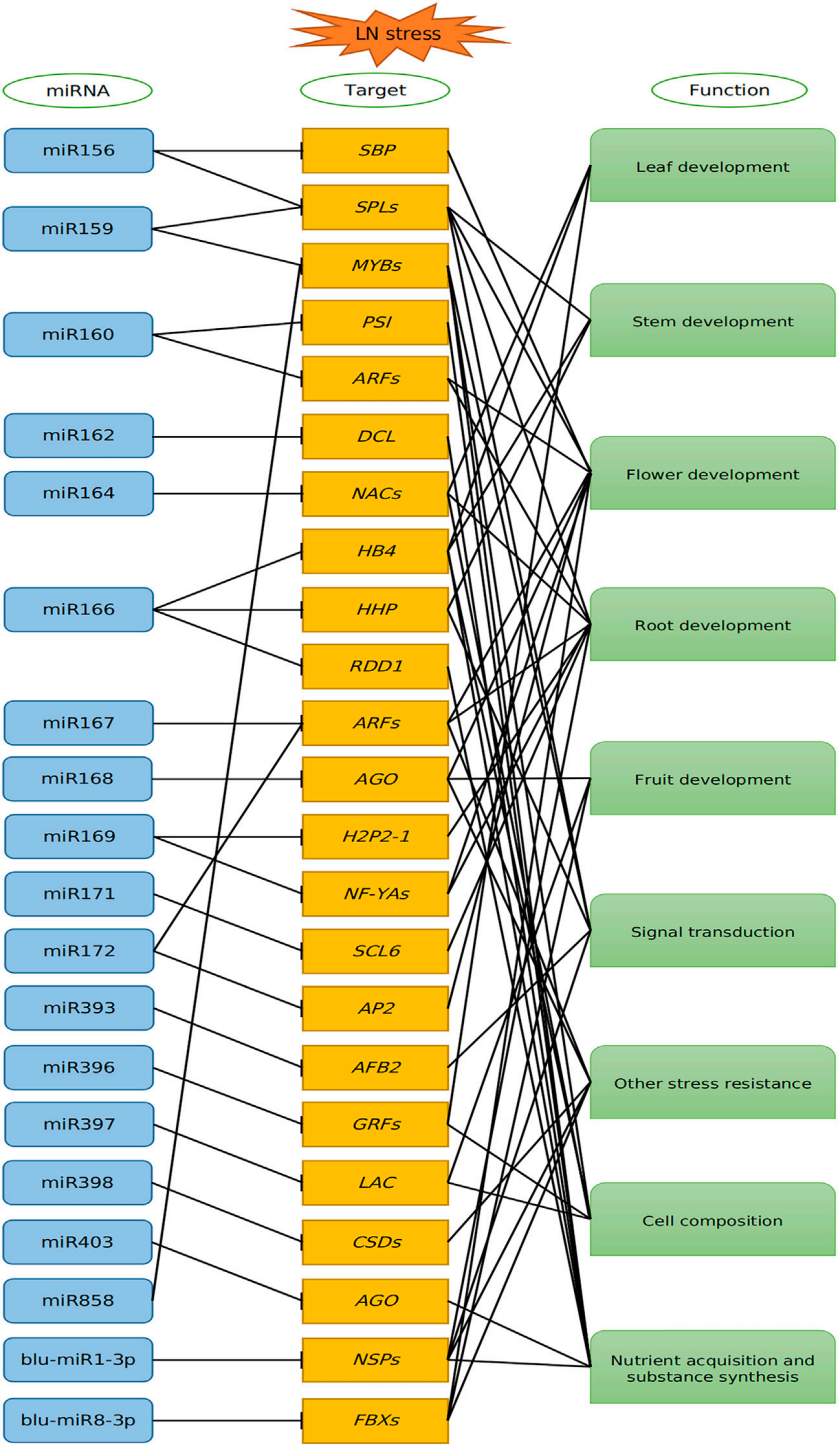
Proposed model of the miRNA-mediated regulatory network in response to low nitrogen in *B luminifera*. The miRNAs negatively regulated their target genes, and these targets are involved in leaf, stem, root development, signal transduction, stress resistance, cell composition and nutrient acquisition, and substance synthesis.

Under LN stress, plants change their phenotypes and transcriptional activity (Curci et al., 2017). Amounting studies showed that miRNAs play essential roles in response to LN stress by cleaving target mRNAs or inhibiting translation of mRNAs. In our study, a series of miRNAs and validated target genes based on degradome sequencing might take part in response to environment starvation; thus, a putative model on miRNA–target interactions under LN stress was proposed (Figure 7). The biogenesis of miRNAs was processed by DCL1, which was regulated by miR162 (Xie et al., 2003). Then, the mature miRNA entered the AGO to regulate target genes. AGO1 was proved to be targeted by miR168, which was verified in this study. In addition, AGO2 was targeted by miR403, as described in our previous study (Pan et al., 2017). Therefore, the miRNA biogenesis and function were feedback regulated by miR162, miR168, and miR403, when the *B. luminifera* encounter the LN stress, thus helping plants to adapt to N starvation condition. In the present study, most of the target genes for known miRNAs were TFs, such as *ARFs*, *HD-Zips*, *NFYAs*, and *SPLs* (Supplementary Table S5), whereas some targets were protein-coding genes, such as Disease resistance protein (RGA), Threonine Synthase (*TS1*) and Laccase (*LACs*). These miRNA–target modules played important roles in transcriptional regulation, auxin response, energy metabolism, biosynthesis, and signal transduction.

The aging pathway in flowering regulation is controlled mainly by miR156. *SPLs* were targeted by miR156, and overexpression of *SPL7* and *SPL8* promotes flowering, and vice versa (Gou et al., 2019). miR156 was induced by LN stress, and the miR156-SPL3/SPL12 module directly activates *MADS50* in the node to regulate crown root development in rice (Shao et al., 2019). In general, miR156 is induced by LN in *Chrysanthemum nankingense* (Song et al., 2015). In our study, miR156s could be divided into two groups based on expression patterns, which was exemplified by the fact that one group was downregulated continuously during the treatment period, whereas the other group was upregulated after 0.5 h treatment and then downregulated after 24 h, which was consistent with the miR156 expression in poplar (Bao et al., 2019). miR156a was repressed under LN stress, and *SPL13* was downregulated after 0.5 h but was upregulated after 24 h of LN stress, which might promote the transition from the vegetative to the reproductive stage under LN stress in *B. luminifera*.

A previous study showed that miR164 played a negative role in adventitious root development by downregulating the expression of *NAC1* (Li J. et al., 2021). A NAC gene *EjNACLl47* had a positive effect on cell expansion and organ enlargement in *Eriobotrya japonica* (Chen Q. et al., 2021). Besides, *NAC* is related to leaf senescence; for instance, *GmNAC81* was upregulated during the leaf senescence, and the overexpression of *GmNAC81* accelerated leaf senescence by accumulating H_2_O_2_ (Ferreira et al., 2020). Leaf senescence is a process of recycling nutrients for other organs (Lim et al., 2007). In the present study, the expression level of miR164 was decreased under LN stress, whereas the target *NAC100* was inhibited after 0.5 h and then was induced significantly after 24 h of LN stress (Figure 6). It suggested that miR164 might play an important role in leaf senescence mainly by targeting *NAC100*, to accelerate the N recycling under LN conditions in *B. luminifera*.

The Class III homeodomain Leu zipper (HD-Zip III) gene family plays essential roles in plant growth and development. For example, overexpression of *HOX32* (an HD-Zip III family member) resulted in curled leaf, reduced leaf angle, semi-dwarf phenotype, and reduced photosynthetic capacity. OsHox32 may regulate plant architecture and leaf development by regulating *YABBY* genes in rice (Li et al., 2016). HD-Zip III including *Hox32* targeted by miR166 and the OsmiR166b-OsHox32 module plays important roles in plant growth and development and plant architecture by regulating the cell wall-related gene expression (Chen H. et al., 2021). However, miR166b-5p was validated to target *HHP1* (Heptahelical protein) in *B. luminifera*. Previous studies showed that *HHP* participated in the signal transduction pathway by cooperating with *MYB* to transduce hormone and environmental signals to cope with cold stress (Lee and Seo, 2015). Therefore, we speculated that the inhibited miR166b-5p to ensure the accumulated target *HHP1* thus took part in the signal transduction pathway to cope with LN stress in *B. luminifera.*

blu-miR1-3p was validated to target *NSP* (nodulation-signaling pathway), an GRAS (GAI, RGA, SCR)-type transcription factor, which was a constituent of the Myc signaling pathway (Delaux et al., 2013). NSP is generally related to nodule symbiosis (Singh and Verma, 2021) and also participated in other stress responses, such as drought stress (Yao et al., 2019). Liu et al. (2011) showed that *NSP1* and *NSP2* are indispensable for strigolactone (SL) biosynthesis both in the legume and rice, which was exemplified by the fact that NSP2 is essential for the conversion of orobanchol into didehydro-orobanchol, dependent on DWARF27, a gene essential for SL biosynthesis (Liu et al., 2011). Therefore, we speculated that novel miRNA blu-miR1-3p might participate in SL biosynthesis by targeting the NSP gene, when *B. luminifera* encounters N starvation. However, the response mechanism of blu-miR1-3p under LN stress still needs to be further studied.

## Conclusion

Integrated analysis of sRNA-Seq and degradome-Seq was conducted to elucidate the molecular mechanisms in response to LN stress in *B. luminifera*. sRNA sequencing identified 98 known miRNAs, and 31 novel miRNAs were differentially expressed after 0.5 or 24 h of LN stress. A total of 122 DEmiRNAs targeted 203 genes, comprising 321 miRNA–target pairs. Thus, a miRNA-mediated network was proposed. Taken together, these findings provide useful information to elucidate miRNA functions and establish a framework for exploring N signaling networks mediated by miRNAs in *B. luminifera* and other woody plants.

## Data Availability

The data presented in the study are deposited in the CNGB Sequence Archive (CNSA) of China National GeneBank DataBase (CNGBdb) repository, accession number CNP0003144.
