# Supplementary material for: Identification of Nitrogen Starvation-Responsive miRNAs to Reveal the miRNA-Mediated Regulatory Network in Betula luminifera
Source: Front Genet. 2022 Aug 17;13:957505. doi: 10.3389/fgene.2022.957505 (PMC9428261; doi:10.3389/fgene.2022.957505)
Supplement: Supplementary file 3 [file Table2.DOCX]

| Table S2 Reads of sRNA liabraries | | | | |
| --- | --- | --- | --- | --- |
| Libraries | Raw reads | Valid reads | Uniq sequence | Valid unique reads |
| Root 0h | 9,997,654 | 2,305,703 | 993,419 | 461,778 |
| Root 0.5h | 16,119,916 | 5,012,446 | 1,854,497 | 1,028,698 |
| Root 24h | 11,902,396 | 2,663,870 | 1,226,137 | 514,935 |
| Shoot 0h | 13,788,646 | 3,475,390 | 1,566,885 | 785,402 |
| Shoot 0.5h | 15,523,889 | 4,953,286 | 2,257,734 | 1,468,323 |
| Shoot 24h | 10,673,847 | 1,322,911 | 1,273,142 | 319,385 |
